# Basic Amino Acid Mutations in the Nuclear Localization Signal of Hibiscus Chlorotic Ringspot Virus p23 Inhibit Virus Long Distance Movement

**DOI:** 10.1371/journal.pone.0074000

**Published:** 2013-09-03

**Authors:** Ruimin Gao, Sek-Man Wong

**Affiliations:** 1 Department of Biological Sciences, National University of Singapore, Singapore, Singapore; 2 Temasek Life Sciences Laboratory, Singapore, Singapore; 3 National University of Singapore Suzhou Research Institute, Suzhou Industrial Park, Jiangsu, China; Virginia Tech, United States of America

## Abstract

The p23 is a unique protein in the Hibiscus chlorotic ringspot virus which belongs to Family *Tombusviridae* Genus *Carmovirus*. Our previous results showed that the p23 is indispensable for host-specific replication and is localized in the nucleus with a novel nuclear localization signal. To investigate additional function(s) of p23, mutations of basic amino acids lysine (K), arginine (R) and histidine (H) that abolish its nuclear localization, were introduced into a biologically active full-length cDNA clone p223 of HCRSV for testing its effects on virus replication and virus movement *in vivo*. Primer-specific reverse transcription-PCR was conducted to detect gene transcript level of *p23* and viral coat protein separately. Virus replication and its coat protein expression were detected by fluorescent in situ hybridization and Western blot, respectively. The effect of p23 was further confirmed by using artificial microRNA inoculation-mediated silencing. Results showed that the two mutants were able to replicate in protoplasts but unable to move from inoculated leaves to newly emerged leaves. Both the *p23* and the *CP* genes of HCRSV were detected in the newly emerged leaves of infected plants but CP was not detected by Western blot and no symptom was observed on those leaves at 19 days post inoculation. This study demonstrates that when p23 is prevented from entering the nucleus, it results in restriction of virus long distance movement which in turn abrogates symptom expression in the newly emerged leaves. We conclude that the p23 protein of HCRSV is required for virus long distance movement.

## Introduction

Among positive-sense single-stranded plant RNA viruses, there are 19 reported members in the genus *Carmovirus*, family *Tombusviridae* (International Committee on Taxonomy of Viruses, http://ictvonline.org/virusTaxonomy.asp? version = 2012). A few of them are studied more extensively. These include Carnation mottle virus [1], Cowpea mottle virus [2], Hibiscus chlorotic ringspot virus (HCRSV) [3], Melon necrotic spot virus [4], Pelargonium flower break virus [5], Saguaro cactus virus [6] and Turnip crinkle virus [7]. In general, the two 5′ proximal open reading frames (ORFs) of *Carmoviruses* encode a p28 and a readthrough p81, which are thought to be involved in virus replication [8]–[10]. The p8 and p9, which are translated from subgenomic (sg) RNA1, are involved in cell-to-cell movement [8], [11]. In addition, coat protein (CP) is also involved in virus movement for TCV [8].

**Figure 1:**
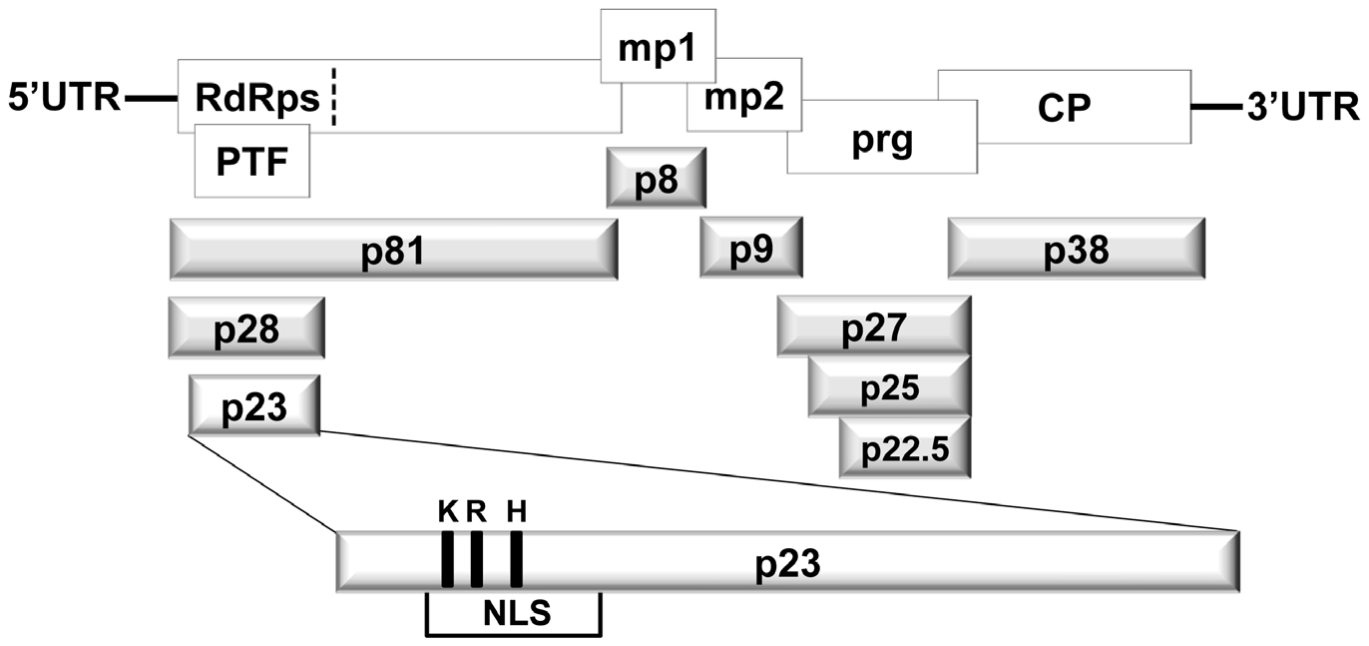
Organization of HCRSV genomic RNA and its corresponding open reading frames and predicted proteins (not drawn to scale). The upper rectangles represent open reading frames. Untranslated region (UTR); putative transcription factor (PTF); RNA-dependent RNA polymerases (RdRps); movement protein (mp); pathogenesis-related gene (prg); coat protein (CP). The dotted vertical line represents a readthrough codon UAG. The 3-dimensional rectangles below represent the corresponding predicted mature proteins. Basic amino acids K, R, and H represent the mutation sites in the nuclear localization signal (NLS) of p23.

HCRSV genome contains 3911 nucleotides with seven ORFs (Figure 1). A biologically active cDNA clone of HCRSV p223 has been obtained previously [3]. The HCRSV CP (p38) [3] is a gene silencing suppressor [12]. In addition, p27 and its other in-frame isoforms (p25 and p22.5) affect symptom expression and potentiate *Carmoviruses* movement in kenaf (*Hibiscus cannabinus* L.) [13]. Different from other *Carmoviruses*, HCRSV contains a novel ORF (p23) which is a putative transcription factor and it is indispensable for host-specific replication [3], [14]. In addition, the p23 possesses a novel nuclear localization signal (NLS) which interacts with importin α and facilitates HCRSV RNA genome to enter nucleus [15].

For the p23 NLS, any mutation to the three basic amino acids lysine (K), arginine (R) and histidine (H) (Figure 1) will abolish its nuclear localization. Since p23 is essential for HCRSV replication and it is a putative transcription factor, whether HCRSV infection can be affected by mutations of the basic amino acids is not known. This study is aimed to address this question and to uncover any additional function(s) of p23, based on mutations of its basic amino acids. This will also contribute to the understanding of virus long distance movement and symptom development.

## Materials and Methods

### Plant Materials and Plasmid Construction

Kenaf seeds (cultivar Everglades 41) were obtained from Mississippi State University, U.S.A. and germinated in potting mixture (Universalerde Universal Potting Soil, The Netherlands) for 7 days. Kenaf seedlings were transferred into potting mixture after emergence of their first true leaves. All plants were grown under 16 h light and 8 h dark conditions at 25°C.

Mutations in the basic amino acids of the p23 NLS, which includes mutants p223 (H to A) and p223 (K, R to A, A), respectively, were introduced into the biologically active full-length cDNA clone of HCRSV p223 [14] using appropriate primers (Table 1). Enzyme DPnI was used to remove the original template after amplification using high fidelity enzyme (KAPA Biosystems) and PCR products were transformed into *Escherichia coli*. Single colonies were picked individually for plasmid preparation and sequence verification.

**Table 1:** Primers used in this study.

Primer	Sequence (5′ to 3′)
I. amiR-p23 F	GATTTTAATTGACTGCACGTCTTTCTCTCTTTTGTATTCC
II. amiR-p23 R	GAAAGACGTGCAGTCAATTAAAATCAAAGAGAATCAATGA
III. amiR-p23* F	GAAAAACGTGCAGTCTATTAAATTCACAGGTCGTGATATG
IV. amiR-p23* R	GAATTTAATAGACTGCACGTTTTTCTACATATATATTCCT
I. amiR-HcSO F	GATTTTAATTGACTGCACGTCTTTCTCTCTTTTGTATTCC
II. amiR-HcSO R	GAAAGACGTGCAGTCAATTAAAATCAAAGAGAATCAATGA
III. amiR-HcSO* F	GAAAAACGTGCAGTCTATTAAATTCACAGGTCGTGATATG
IV. amiR-HcSO* R	GAATTTAATAGACTGCACGTTTTTCTACATATATATTCCT
A	CTGCAAGGCGATTAAGTTGGGTAAC
B	GCGGATAACAATTTCACACAGGAAACAG
P23F	CCGGAATTCATGCTTTCTCAATTGCTTTC
P23R	CGCGGATCCCGGGCGAGTACCCCTGAAA
H-CP-F	CTGAATTCCATGCTGCAGAAGAATGACC
H-CP-R	GCGGATTC CTAGTTCCTACAGGCCCAC
P23(K,R-A,A)58–123F	CGCAGCTGTTGGAATTATCAGACACCCCCGTATCGCTCCACGATGA
p23(K,R-A,A)58–123R	CGGGGGTGTCTGATAATTCCAACAGCTGCGGCGAAAGGTGCGACACA
p23 (H-A)58–123F	AATTATCACGCACCCCCGTATCGCTCGACGAGATCCTTGCATCGCTG
p23 (H-A)58–123R	AAGGATCTCGTCGAGCGATACGGGGGTGCGTGATAATTTCAACAGCTG
HcAct-qF603	ACGAGCAGGAACTGGAGACT
HcAct-qR734	TGAGTGATGGCTGGAAGAGGA

Artificial-microRNA (amiRNA) amiRp23 or amiRSO was engineered into the miR319a precursor (plasmid pRS300) by site-directed mutagenesis (overlapping PCR) (Table 1), following the protocol described by Rebecca Scheab of Max-Plank Institute for Developmental Biology, Tuebingen, Germany (2005) (http://wmd.weigelworld.org/cgi-bin/mirnatools.pl. Ossowski Stephan, Fitz Joffrey, Schwab Rebecca, Riester Markus and Weigel Detlef, personal communication). The amiRp23 or amiRSO fragment was inserted into pGreen vector with *EcoRI* and *BamHI* restriction enzyme sites. The verified plasmid was transformed into *Agrobacterium tumefaciens* GV3101 using electroporation and the transformed colonies were verified by colony PCR.

### Plant Inoculation with *in Vitro* Transcripts of p223 and its Two Mutants

Plasmids (p223, p223 (H to A) and p223 (K, R to A, A) were linearized with *SmaI* and transcribed accordingly using the *in vitro* transcription kit (Ambion, mMESSAGE mMACHINE). *In vitro* transcribed RNA was verified for integrity by gel electrophoresis. One µg of the transcribed RNA was mixed with equal volume of 2×GKP buffer (50 mM glycine, 30 mM K_2_HPO_4_, pH 9.2, 1% bentonite and 1% celite). Two fully expanded true leaves from healthy kenaf plant were inoculated. Twelve individual plants were used for each treatment. In addition to plants treated with *in vitro* transcribed RNAs of the wild type (wt) HCRSV (positive control) and two mutant viruses, plants were also inoculated with 1 × GKP buffer as mock (negative control). The experiments were repeated twice.

### Preparation of Kenaf Protoplasts for Fluorescent in Situ Hybridization (FISH)

Protoplasts were isolated following previously described protocol [16]. Briefly, the collected kenaf leaves were sterilized for 10 min with 0.8% (v/v) Clorox® and cut into 1 mm×1 mm stripes and then digested with filter-sterile cellulase (0.8%) and macerase (0.25%) for 16 h. The isolated protoplasts were transfected with *in vitro* transcribed RNA from full-length HCRSV wt and its basic amino acid mutants. At 72 h post transfection, protoplasts were concentrated by 100×*g* centrifugation and fixed with the fixation buffer 4% (w/v) paraformaldehyde, 2.5% sucrose (w/v) in 0.1 M phosphate buffer (pH 7.2) for 2 h at room temperature. The FISH procedures were adopted from a protocol described by Zenklusen and Singer [17], [18] with slight modifications. Briefly, 50 ng Cy3-labeled HCRSV cDNA probe [bind to genomic RNA (gRNA) nt 350 to 301 which is located in the region of the *p23* sequence]. The probe sequence 5′-GTTGGAGTGCCCCCAAAATGTAGCTTTGCTGCGTTGCCGCATGGAGAGC-3′ was used to localize HCRSV RNA. All fixed protoplasts were attached to poly-L-lysine coated coverslips and stored in 70% ethanol (v/v) at −20°C before hybridization. Coverslips were inverted onto 25 µl of hybridization solution containing 50 ng of Cy3 labeled DNA probe in solution F (40% formamide, 2×SSC, 10 mM NaHPO_4_, pH 7.5) and solution H (2×SSC, 2 mg/ml BSA), 1 µg yeast tRNA and 1 µg salmon sperm DNA and hybridized overnight at 37°C. Finally, the fixed protoplasts on the coverslips were stained with DAPI and mounted with Vectashield® medium. Images were obtained using a confocal laser scanning microscope (Carl Zeiss LSM510 META confocal, Germany). Three-dimensional images of protoplasts were captured and the average density of signals was quantified using Velocity 6.1.1 software (PerkinElmer).

### RNA Extraction and cDNA Synthesis for RT-PCR and qRT-PCR

Both inoculated and newly emerged leaves were harvested for RNA extraction with TRIzol® reagent (Invitrogen) at 19 days post inoculation (dpi). Total RNA (∼2 µg) were used to generate cDNAs through reverse transcription, using oligo(dT)_15_ as primer and SuperScript® III Reverse Transcriptase kit (Invitrogen). Reverse transcription PCR (RT-PCR) (Table 1) was used to analyze the gene transcript levels of *p23* and *CP*. The quantitative real-time reverse transcription PCR (qRT-PCR) was used to analyze amiRp23 expression with the CFX384™ real-time PCR detection system (Bio-Rad). *Actin* gene was amplified using appropriate primers (Table 1) as an internal control for all qRT-PCR. Samples of mock, HCRSV wt and two mutants included three biological repeats and each qRT-PCR sample provided three technical repeats. The qRT-PCR results from different treatments were subjected to the Student’s *t*-test for statistical analysis.

### Western Blot Analysis of HCRSV CP

Western blot was carried out according to previously published protocol [19]. Briefly, inoculated and newly emerged leaves (0.1 g) from p223, p223 (H to A) and p223 (K, R to A, A) were collected for protein extraction, with 0.2 ml of protein extraction buffer [220 mM Tris–HCl, pH 7.4, 250 mM sucrose, 50 mM KCl, 1 mM MgCl_2_, 2 mM phenylmethylsulfonyl fluoride, 10 mM β-mercaptoethanol, and 1×complete EDTA-free protease inhibitor (Sigma, St. Louis, USA)]. The denatured protein samples were separated on the 12% SDS PAGE gel and transferred onto a nitrocellulose membrane (Bio-Rad), followed by incubation with anti-HCRSV antibody and visualized using nitroblue tetrazolium/5-bromo-4-chloroindol-3-yl phosphate (Fermentas).

### *Agrobacterium Tumefaciens*-Mediated Transient Expression of amiRp23 and amiRSO

*Agrobacteria* liquid culture containing each of the pGreen-amiRp23, pGreen-amiRSO (a negative control, it targets a host factor sulfite oxidase) and 35SpGreen plasmids (a second negative control), was grown to OD reading (at 600 nm) between 1.0–1.5 and harvested. The cell pellet was resuspended in infiltration buffer (pH 7) containing 10 mM each of MgCl_2_ and 2-(N-morpholino) ethanesulfonic acid (MES), and 100 µM acetosyringone and infiltrated into kenaf leaves using a syringe without needle [13]. The experiment was carried out thrice.

### Inoculation of amiRp23 and amiRSO into Apical Meristems of HCRSV-infected Kenaf Leaves

Inoculation procedures followed the published protocols [20], [21] with slight modifications. Briefly, *Agrobacteria* were resuspended in the infiltration buffer (1×10^8^ cell/ml) instead of water and used as inoculum. After pricked with a needle (*Ф* 0.71 mm), the apical meristems of 10-day-old seedlings (10 cm height) were inoculated with a cotton applicator drenched with the inocula or infiltration buffer alone (as a mock treatment). The inoculated seedlings were kept at 22°C in the dark for 3 days and subsequently grown under 16 h light and 8 h dark conditions at 25°C. After one month, the newly emerged leaves were observed for symptom expression.

## Results

### Viral Replication was Unaffected in the Two HCRSV Mutants

**Figure 2:**
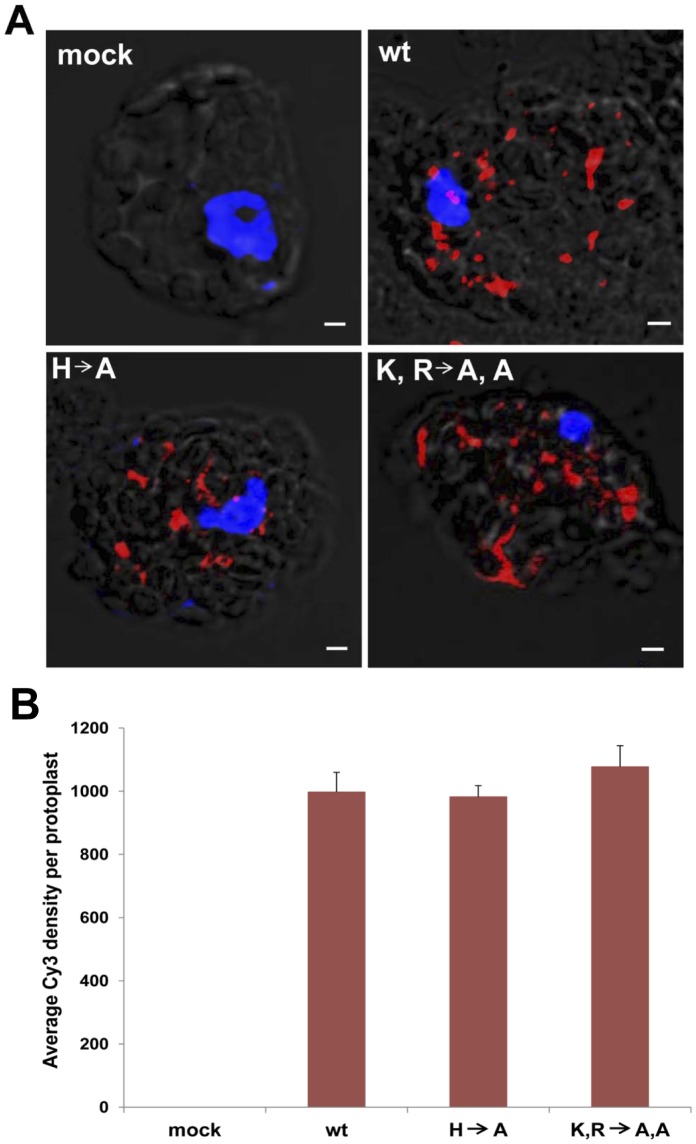
Comparative replication of HCRSV wild type (wt) and two p23 mutants. (A) A single molecule fluorescent *in situ* hybridization (FISH) method was used to detect virus replication, using a Cy3-labeled cDNA probe (corresponding to the HCRSV *p23* coding region at nt 350–301). DAPI stained nuclei (blue-color foci) were superimposed with the differential interference contrast to form a merged image. Kenaf protoplasts were fixed with 4% paraformaldehyde. Cy3 signals were not detected in mock transfected protoplasts. Representative sections for each of the three dimensional images of protoplasts transfected with HCRSV wt and two mutants were shown (mock, wt, H to A and K, R to A, A). Single RNA molecules (red dots) were detected in the protoplasts transfected with *in vitro* transcript of full-length cDNA clone of HCRSV wt or mutants H to A (H A) or K, R to A, A (K, R A, A ) mutant at 72 hours post transfection. The average density of Cy3 signals in the protoplasts quantified using Velocity 6.1.1 software (PerkinElmer) showed that the replication level was similar for the HCRSV wt and its two mutants. Bar = 2 µm. (B) Comparison of the average Cy3 density measured from 10 protoplasts for mock, HCRSV wt, HCRSV basic amino acid mutants H to A and K,R to A,A, respectively. Standard deviations were shown.

The three basic amino acid mutations of p23 also resulted in changes of both amino acids tryptophan (T) and proline (P) to arginine (R) in the p28 and thus its readthrough protein p81 which encode the putative RNA-dependent RNA polymerases. Therefore, we first investigated if the replication of these two mutants were affected due to changes in the p28/p81. Using the Cy3-labeled probe with the FISH method, we monitored the viral replication of the HCRSV wt and the two mutants in kenaf protoplasts. RT-PCR results showed that basic amino acids lysine (K), arginine (R) and histidine (H) remained in their mutated status as alanine (A) within 72 hours post transfection. A representative section for each of the three dimensional images of protoplasts transfected with HCRSV wt and two mutants was shown (Figure 2A). Results showed that no Cy3 signal was observed in the mock transfected kenaf protoplasts. There was no difference in the density of red dots (indicating the presence of viral gRNA) between HCRSV wt and the two mutants (Figure 2A). The replication level of the HCRSV wt and the two mutants was similar, as quantified by the average density of Cy3 signals present in the protoplasts (Figure 2B). These results indicated that virus replication was not affected by the basic amino acid mutations introduced into the NLS region of p23.

### Symptoms were only Observed in HCRSV wt-inoculated Kenaf Leaves at 19 dpi

**Figure 3:**
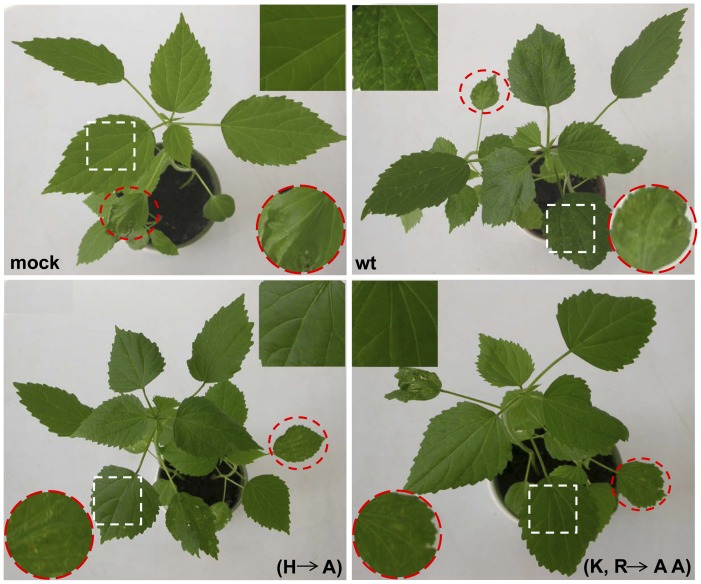
Comparison of HCRSV symptoms on kenaf leaves infected with wild-type (wt) virus and its two mutants at 19 days post inoculation. HCRSV symptoms were not observed in the mock control. HCRSV wt infected leaf displayed chlorosis. Symptoms were observed in the inoculated leaves (red circles with dotted outline) but not in the newly emerged leaves (white squares with dotted outline) of p223 (H A) and p223 (K, R A, A) mutants. Close-ups of newly emerged leaves and inoculated leaves are shown in inserts with squares and circles, respectively.

After observing that the replication of the two virus mutants was not affected, *in vitro* RNA transcripts of these two mutants were inoculated onto kenaf plants. Since severe symptoms were observed on the HCRSV wt infected plants at 19 dpi, symptoms caused by HCRSV wt and two mutants were chosen for investigation at this particular time point. No symptom was observed in the inoculated and newly emerged leaves of mock inoculated plants (Figure 3, mock, white square and red circle with dotted outlines), only some mechanical damage due to inoculation was observed (mock, white square with dotted outline). Chlorosis was observed on the inoculated and newly emerged leaves of HCRSV wt infected plants (Figure 3, wt, white square and red circle with dotted outlines). In both virus mutants p223 (H to A) and p223 (K, R to A, A) inoculated plants, chlorotic spots were observed only on the inoculated leaves but not on the newly emerged leaves (Figure 3, H to A; K, R to A, A; white squares and red circles dotted outlines).

### Detection of *p23* and *CP* Transcript Level in the Newly Emerged Leaves of Kenaf Plants Inoculated with HCRSV wt and its Two Mutants at 19 dpi

**Figure 4:**
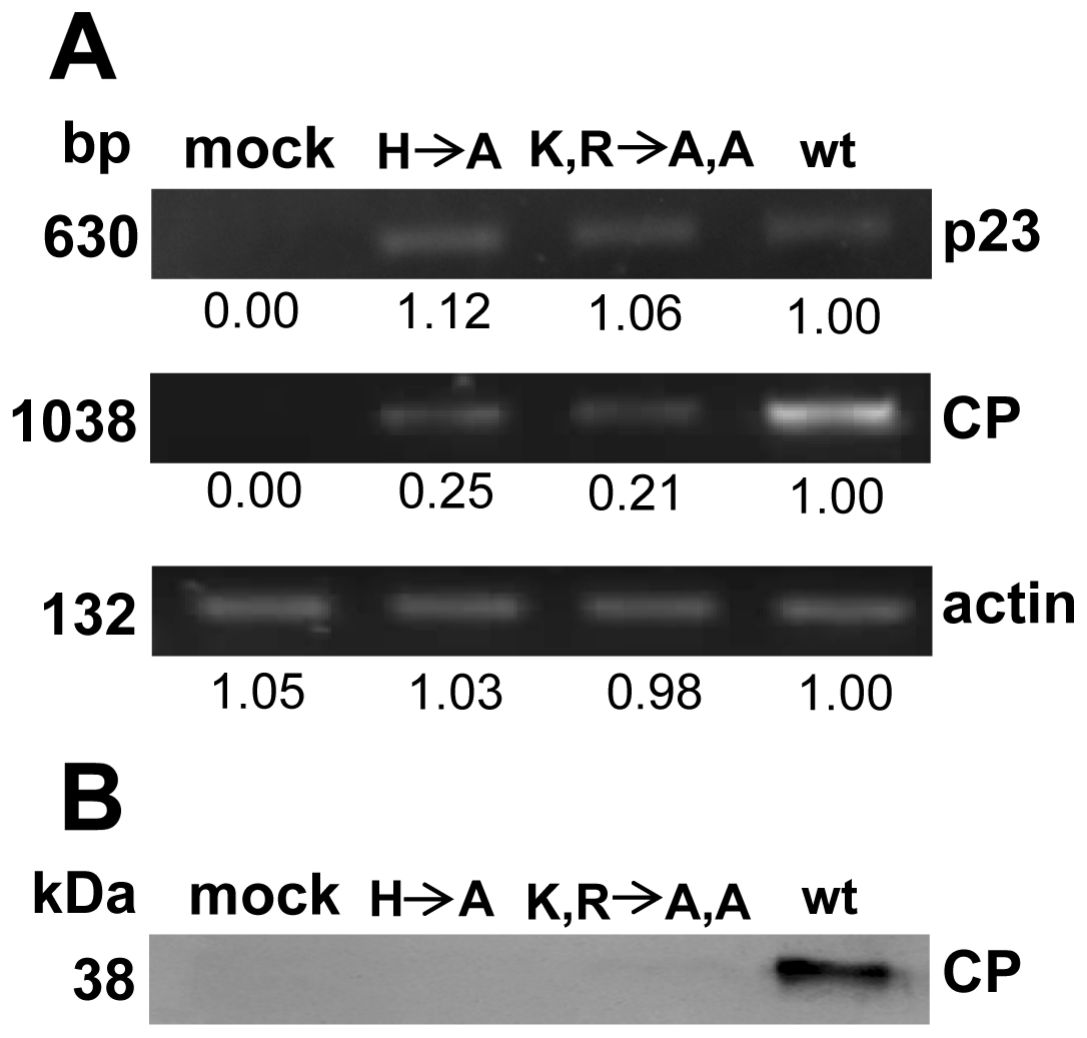
Detection of *p23* and *CP* of HCRSV in newly emerged wild type (wt), (H to A) and (K, R to A, A) inoculated kenaf plants at 19 days post inoculation (dpi), respectively. (A) Both *p23* and *CP* genes were detected in the newly emerged leaves of plants infected with two mutants (H A) and (K, R A, A) at 19 dpi using RT-PCR. *Actin* gene was used as a loading control. The band intensity of each gene transcript from RT-PCR results was quantified using ImageJ. (B) Detection of HCRSV-CP protein in the wt, mutants H to A and K, R to A, A in the newly emerged kenaf leaves at 19 dpi. The CP expression level was below detection level for the two mutants.

In addition to visual comparison of symptoms, RT-PCR was performed to investigate the expression of *p23* and *CP* genes. The p23 ORF is located at the 5′-end of the gRNA and is translated directly from the positive single-stranded gRNA. Consequently, the RT-PCR would not only be able to monitor the amount of *p23* gene transcript but it would also monitor the total amount of replicated (+) and (–) strand gRNAs in both inoculated and systemically infected leaves. The results showed that *p23* gene transcript was detected from newly emerged leaves of HCRSV wt and two mutants p223 (H to A) and p223 (K, R to A, A)-inoculated kenaf plants at 19 dpi. The *actin* gene was used as a loading control (Figure 4A). Sequencing results of the RT-PCR products derived from newly emerged leaves at 19 dpi showed that for the first mutant p223 (H to A), the alanine (A) was reverted back to histidine (H). For the second mutant p223 (K, R to A, A), one alanine (A) was reverted back to lysine (K) and the second alanine (A) was not reverted back to arginine (R).

The *CP* gene is located at the 3′-end and is therefore also present in the gRNA. Thus, the RT-PCR results using the *CP* primer set is able to monitor the content of the gRNA and two sgRNAs in both inoculated and systemically infected leaves. The results showed that *CP* gene was detected in the newly emerged leaves of plants inoculated with HCRSV wt and its two mutants p223 (H to A) and p223 (K, R to A, A) at 19 dpi (Figure 4A). However, the amount of *CP* gene expression was insufficient to be detected (Figure 4B). There was absence of viral symptoms in the newly emerged kenaf leaves infected with the two mutants at 19 dpi (Figure 3, squares with dot outlines).

### Less Severe Symptoms in pGreen-amiRp23-inoculated Kenaf Plants Pre-inoculated with HCRSV

**Figure 5:**
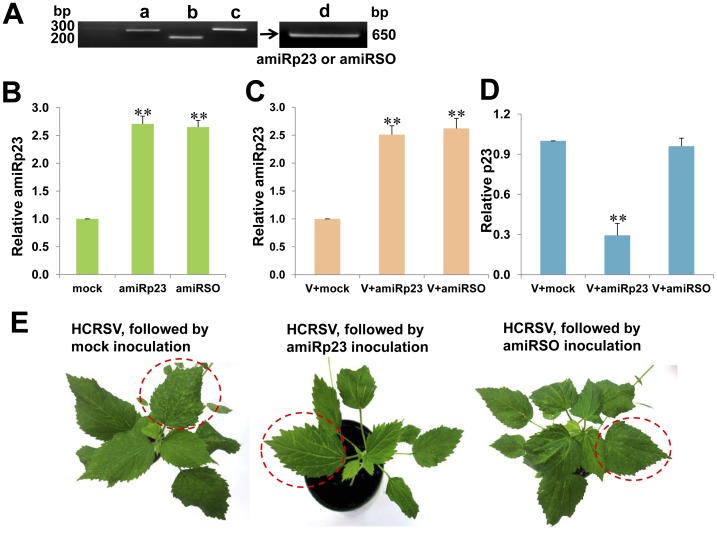
Less severe symptoms were observed in the newly emerged leaves from amiRp23-inoculated kenaf plants which were pre-infected with HCRSV 10 days earlier. (A) Overlapping PCR to obtain amiRp23 or amiRSO. The a, b, c represent the required fragments to form amiRp23 or amiRSO. PCR fragment d is the product of overlapping PCR from a, b and c. (B) Verification of amiRp23 and amiRSO transient expression in the kenaf leaves using *Agrobacterium*-infiltration by qRT-PCR. Mock, amiRp23 and amiRSO represent total RNA from mock, amiRp23 and amiRSO *Agro*-infiltrated kenaf leaves, respectively. Verification of amiRp23 (C) and *p23* gene (D) transcript levels in the HCRSV-infected kenaf leaves treated with mock, amiRp23 and amiRSO by qRT-PCR, respectively. V+mock, V+amiRp23 and V+amiRSO represent total RNA from HCRSV infected leaves followed by inoculation with mock, amiRp23 and amiRSO, respectively. (E) Comparison of symptoms (red circles with dotted outline) in the newly emerged leaves between mock (more severe) and amiRp23-inoculated kenaf leaves (less severe) pre-inoculated with HCRSV 10 days earlier, using visual observations. Significant differences of relative amiRp23 and *p23* gene between mock with amiRp23 and mock with amiRSO treated plants were calculated using the one sample Student’s *t*-test. Asterisks (∗∗) indicate significance at 0.01 levels of confidence.

In order to verify if p23 plays a vital role in the virus long distance movement and resulted in altered symptom expression in the newly emerged leaves, an amiRp23 which targets p23 was synthesized. In addition, a negative control amiRSO which targets a host factor sulfite oxidase and possesses a different seed region as amiRp23 was included. The three PCR fragments of a, b, and c, with respective lengths of 289 bp, 209 bp and 316 bp, were obtained (Figure 5A). The overlapping PCR product d was 694 bp in length (Figure 5A). The amiRp23 or amiRSO fragment inserted into a pGreen vector to form pGreen-amiRp23 or pGreen-amiRSO construct was obtained. Firstly, amiRp23 or amiRSO was transiently expressed in the infiltrated kenaf leaves (Figure 5B) to test whether amiRp23 and amiRSO can be overexpressed *in vivo*. In order to study the effect of amiRp23 affecting HCRSV symptom development, generation of putative transgenic plants expressing amiRp23 were attempted using the *Agrobacterium*-mediated transformation method previously described [20], [21]. The amiRp23 or amiRSO transcript was detected at 30 dpi (Figure 5C), similar to the results obtained from the transient expression experiment (Figure 5B). Silencing efficiency of amiRp23 was also monitored in the HCRSV-infected plants and HCRSV-infected plants, followed by mock, amiRp23 or amiRSO inoculation (Figure 5D). There were significant differences of relative amiRp23 and *p23* gene between mock with amiRp23 and mock with amiRSO treated plants at 0.01 levels of confidence, using the one sample Student’s *t*-test (Figure 5B, C, D). Symptoms were less severe in the HCRSV-infected plants which were Agro-inoculated with pGreen-amiRp23, as compared to the plants inoculated with buffer alone or pGreen-amiRSO (Figure 5E).

## Discussion

Previously by using *Agrobacterium*-mediated transient expression of p23-GFP fusion protein, we have shown that any single or combinations of mutations of the three basic amino acids in the NLS of p23 would abolish its nuclear localization *in vivo* [15]. In this study, two representative mutants p223 (H to A) and p223 (K, R to A, A), were chosen to test the effects of basic amino acids on infected kenaf plants. The first mutant p223 (H to A) has one basic amino acid mutated from histidine (H) to alanine (A), and the second mutant p223 (K, R to A, A) has two basic amino acids mutated to alanine (A). We investigated the effects of these two mutants on virus movement. From these results, we extrapolate the same effects to other basic amino acids within the NLS of p23 mutants on virus movement. In order to determine the subcellular localization of p23 protein, a GFP fused protein driven by the 35S CaMV promoter was used [15]. The GFP signal in the fused protein can be traced using confocal laser microscopy. However, in the virus mutants, localization of p23 protein is not possible due to its minute amount produced. Since p23 is an individual ORF expressed in the virus mutant with the same amino acids as in the GFP fused protein, it is reasonable to believe that mutations in the p23 region will also yield similar results when there are expressed as part of the virus mutants.

The replicase of RNA viruses, except retroviruses, is highly error-prone [22]. Generally artificially introduced mutations in a virus genome will be reverted back under selection pressures. As a result, RNA viruses can rapidly eliminate genetic mutations introduced into their genomes. However, mutations may induce certain phenotypes on the infected plants. In this study, no symptom was observed in the newly emerged leaves of kenaf plants infected with the two mutants at 19 dpi.

In addition to the NLS of p23, we have also identified the HCRSV gRNA in the nucleus where one of the predicted viral miRNAs (vir-miRNAs) hcrsv-miR-H1-5p is thought to be generated from the p23 sequence [15]. Although the two mutations in the basic amino acids in p23 did not affect virus replication (Figure 2), they could disrupt the nuclear localization of p23 and prevent the entry of HCRSV genome into the nucleus. It is believed that vir-miRNAs play essential roles in overcoming host resistance for efficient infection of viruses [23]. When the p23 is unable to enter nucleus, it will not be able to interact with host genomes and thus interferes with host transcription that confers resistance. In addition, the lack of vir-miRNAs generated by the *p23* sequence would not be made available to target host sequences to counteract host defence.

In this study, we have adopted two approaches to uncover additional function(s) of p23. First, a transgenic approach was used to study the function of amiRp23 to silence its target *p23* gene. The amiRp23 was designed specifically to cleave p23 messenger RNA or to inhibit p23 translation. It may also downregulate viral gRNA in the *p23* region, but not other regions of the viral genome. The sgRNAs and viral genes located outside the *p23* gene region are not affected. Therefore, the downregulation of *p23* using amiRp23 (Figure 5D) caused silencing of *p23* and inhibition of long distance virus movement (Figure 3) and resulted in reduced symptom severity in infected leaves (Figure 5E). Since the kenaf plants grown under our laboratory conditions did not produce flowers, we were unable to generate transgenic progeny plants. However, using the apical meristem inoculation method, we have introduced amiRNA into the plants to silence *p23*. Due to these limitations, we were also unable to generate a reporter transgene to monitor the silencing efficiency of amiRp23. Another approach is the use of the single molecule detection technique FISH to study virus replication. It is a direct visualization technique which tracks signals in a single cell. It is a more specific and sensitive method for study of viral replication, as compared to Northern blot which requires relatively larger amount of RNA samples. We chose this method over northern blot due to the lack of sufficient amount of RNAs present in some of our test samples. Therefore, the FISH technique can be broadly applied to other virus replication studies.

In conclusion, this study has uncovered an additional function of p23 of HCRSV. Although the basic amino acid mutants of p23 are prevented from entering the nucleus, those mutants are still able to replicate but unable to be moved long distance. This indicates yet another example of multi-functional roles of plant viral genes.
